# Association between Occupational Stress and Respiratory Symptoms among Lecturers in Universiti Putra Malaysia

**DOI:** 10.5539/gjhs.v4n6p160

**Published:** 2012-09-28

**Authors:** Nur Aqilah M. Y., Juliana J.

**Affiliations:** 1 Department of Environmental and Occupational Health, Faculty of Medicine and Health Sciences, Universiti Putra Malaysia, Serdang, Malaysia

**Keywords:** High strain, lecturers, occupational stress, respiratory symptoms, Universiti Putra Malaysia

## Abstract

There was considerable evidence that a subject’s psychological status may influence respiratory sensations and that some subjects may experience respiratory symptoms regardless of the presence of a respiratory disease. The objective of this study was to determine the association between occupational stress and respiratory symptoms among lecturers. This cross sectional study was conducted in Universiti Putra Malaysia, involved 61 lecturers from various faculties. Job Content Questionnaire (JCQ) and questionnaires based on American Thoracic Society were used to collect the data on socio-demography, stress level and respiratory symptoms. High level of occupational stress (high strain) was determined among 16 of the respondents (26.2%). Breathlessness was the common symptom experienced by the respondents. Female lecturers were significantly experienced high stress level compared to male (p=0.035). They were also significantly having more breathlessness symptom compared to male lecturer (p=0.011). Study highlighted in study population, gender plays a significant role that influenced level of occupational stress and also gender has role in resulting occupational stress level and respiratory symptoms. There was no significant association between occupational stress and respiratory symptoms. It can be concluded that this group of lecturers of Universiti Putra Malaysia did not experienced high occupational stress level. Occupational stress level was not statistically significantly associated with all respiratory symptoms being studied.

## 1. Introduction

Occupational stress was a complex biopsychosocial situation and has been recognized as major health hazard for employees (Sun et al., 2011). According to Salo (2002), stress comes from imbalanced situations where the demands on an employee exceed or undervalue the employee’s actual conditions, or situation, where needs and goals were continually frustrated. In an organizational context, occupational stress was also known as job stress and/or work stress (Azman et al., 2009). WHO (2011) described work-related stress as the response people may have when presented with work demands and pressures that are not matched to their knowledge and abilities and which challenge their ability to cope.

Researches reveal that teaching profession was an occupation with high risk of stress, and the working conditions in schools place teachers at high risk for burnout (Shun, 2009). Kyriacou et al. (2000) stated that teacher stress can be defined as experience of teacher of unpleasant negative emotions, such as anger, frustration, anxiety, depression and nervousness resulting from some aspect of their work as educator. Stansfeld *et al.*, (2004) reported that educators are at the highest rates of feeling that their jobs are “very stressful” or “extremely stressful”. In a cross-sectional study conducted among 580 secondary school teachers in Kota Bharu District, prevalence of stress was reported as 34% with 17.4% experienced mild stress (Azlihanis *et al.*, 2006). Academic university lecturers in China suffer a rather serious occupational stress where the average raw score of Perceived Stress Questionnaire was 91.0% which is 4.7% and 9.8% higher than the levels obtained from doctors and teachers in primary and high schools respectively (Sun et al., 2011).

Respiratory symptoms were the manifestation of disturbance of our respiratory system. It might be caused by multiple factors in the surrounding. In a study conducted by Cohen et al. (1999), eight respiratory symptoms related to work were measured which were sneezing, nasal discharge, nasal congestion, sore throat, cough, malaise, headache and chilliness. A pilot survey conducted among elementary school staff in Massachuset also study the four respiratory symptoms related to work which were wheezing, coughing, chest tightness and shortness of breath (Deval et al., 2007). According to previous study, there was significant evidence that stress heightened the respiratory rate. While someone was stressed the body needs more oxygen from the environment. To do this, the body attempts to elevate the respiratory rate, which helps to gather more oxygen. This usually causes breathlessness and some other respiratory problem (Hing et al., 1995).

Psychological or environmental stress was not described as a risk factor for respiratory symptoms in most recent reviews. However, there have been reports showing a relationship of stress to streptococcal throat infections, upper respiratory tract infection in premenstrual women, experimentally induced rhinovirus respiratory infections and acute respiratory infections in children (Graham et al., 1986). In other study by Wright et al. (2010), war-related stressors were associated with elevated risk of incident asthma in elderly Kuwaiti civilians exposed to the 1990 Iraqi invasion and occupation.

Nowadays, university teachers are experiencing high level of stress and it was proven by many study and researches. Several classical studies that relate stress or psychological factors with respiratory symptoms have been conducted in another countries and the result showed was quite significant (Cohen et al., 1991; Jacobs et al., 1970). In a report by Safety and Health Executive United Kingdom, Table 2c, it was reported that there was a high rates of self-reported stress, depression and anxiety in the education sectors. Then, in Table 2b, teachers in the Bristol Study report the highest rates of feeling their jobs were ‘very stressful’ or ‘extremely stressful’ (Stansfeld et al., 2004).

**Table 1:** Socio-demography information (N=61)

Variables	Category	N (%)
**Age**	28 – 44	29 (47.5)
	45 - 61	32 (52.5)
**Gender**	Male	25 (41.0)
	Female	36 (59.0)
**Race**	Malay	53 (86.9)
	Chinese	2 (3.3)
	Indian	3 (4.9)
	Others	3 (4.9)
**Marital status**	Single	4 (6.6)
	Married	54 (88.5)
	Divorced/Separated	1 (1.6)
	Widowed	2 (3.3)
**Numbers of family members supported**	0	11 (18.0)
	1	9 (14.8)
	2	11 (18.0)
	3	12 (19.7)
	4	6 (9.8)
	5	5 (8.2)
	6	5 (8.2)
	7	1 (1.6)
	9	1 (1.6)
**Educational level**	Degree	1 (1.6)
	Master/PhD	60 (98.4)
**Working experience**	4 - 14	34 (54.8)
	15 - 25	16 (25.8)
	26 – 36	9 (14.5)
	37 - 47	2 (3.2)

**Table 2:** Descriptive analysis of stress factors studied

Stress factors	Mean±SD	Range
Decision latitude	72.26±6.287	56 - 84
Psychological job demand	34.77±5.780	14 - 48
Job insecurity	4.38±1.227	3 - 8
Social support	23.08±2.557	18 - 31

This study was conducted to evaluate occupational stress level experienced by lectures in Universiti Putra Malaysia and to know whether if there was an association with respiratory symptoms. The symptoms are cough, phlegm, episodes of cough and phlegm, wheezing, breathlessness, chest cold and chest illnesses. Universiti Putra Malaysia was well-known as one of Research University in Malaysia and researches were mainly conducted by the professors and lecturers. While pursuing with the researches, in the same time, these people need to fulfill the requirement of teaching and managing their students. Thus, their responsibility become wider and stress might come along in their daily life.

Thus, it was important to conduct this study as it aims to evaluate the association between occupational stress and respiratory symptoms. This study was concentrated on work-related and socio-demography factors that influenced occupational stress level and its association with respiratory symptoms experienced by the respondent. Indirectly, this study will revealed how high the level of stress experienced by lecturers in Universiti Putra Malaysia and suggestion could be made to lessen stress level and improve their quality of health. Besides that, this study would be a preliminary study that study on the association between occupational stress and respiratory symptoms among lecturers in Malaysia.

## 2. Methodology

### 2.1 Study Background

This cross sectional study was carried out in Universiti Putra Malaysia (UPM). The university was established in 1931 and consists of 16 faculties and 9 institutes. The study population of this study was all UPM lecturers with working experience more than 2 years, non smoking and without respiratory diseases. Sampling frame was lists of male and female lecturers from 8 faculties obtained from administrative office or official website of the faculties. Sample size was 136 where it represents 30% lecturers from each 8 faculties. Respondents were selected by multi-stage random sampling technique.

### 2.2 Determination of Occupational Stress Level and Respiratory Symptoms

Job Content Questionnaires (JCQ) was used to obtain data on socio-demography such as age, gender, ethnicity, education level, marital status, numbers of family members supported, working experience, smoking habits and disease history. Data on decision latitude, psychological job demand, social support and job insecurity which contribute to occupational stress level was also obtained by using this questionnaire. Reliability was determined for internal consistency using Cronbach’s alpha coefficients on Malay version of JCQ by Azlihanis et al. (2006), in her research among secondary school teacher. Cronbach’s alpha for decision latitude was 0.75, psychological job demand was 0.50, social support was 0.84 and job insecurity was 0.65. The study also demonstrated that scales of JCQ were reliable and valid for assessing the psychosocial work conditions. Respiratory symptoms were obtained using questionnaires adapted from American Thoracic Society. Pre-test was performed on 10% of the sample size to verify validity of the questionnaires.

### 2.3 Data Analysis

Statistical analysis was carried out using Statistical Packages for Social Sciences (SPSS version 20). Categorical variables were presented as frequencies and percentages. Chi-square test and Fisher’s Exact Test were used to make comparison and to determine association between categorical variables. Results of the association were also expressed as Odds Ratio (OR) and 95% confidence interval. Continuous variables were presented as mean and standard deviation (SD). A two-sided p-value less than 0.05 was considered statistically significant.

## 3. Results

### 3.1 Response Rate and Socio-demography Information

A total number of 136 lecturers have been informed that they were randomly selected to be involved in the study. Questionnaires have been distributed to all the respondents and time has been given for them to answer the questionnaires. Numbers of questionnaires that have been returned were 61 which consist of 25 male and 36 female respondents. The response rate was 44.9% which indicate less than half of the respondents responded to the study. Table 1 shows frequencies and percentages of socio-demography data collected. Age of the respondents was in range between 28 until 61 years old. 29 respondents (47.5%) fall under the first age category which was from 28 to 44 years old. Another 32 respondents (52.5%) were in the range of age from 45 to 61 years old. Out of 61 respondents, 41.0% were male. The majority (86.9%) were Malays, 88.5% of the respondents were married. Most of the respondents supported 3 or less family members and possessed Master/PhD as their highest education level. Majority of the respondents had worked as lecturers in the range from 4 to 14 years.

### 3.2 Occupational Stress Level

Determination of occupational stress level was obtained by considering 4 factors which were decision latitude, psychological job demand, job insecurity and social support. Table 2 shows descriptive analysis of the 4 factors being studied. Frequencies and percentages of respondents with high and non-high stress level were showed in Table 3. 26.2% respondents experienced high occupational stress level.

**Table 3:** Occupational stress level (N=61)

Stress level	N (%)
High strain	16 (26.2)
Low strain	14 (22.9)
Passive group	18 (29.5)
Active group	13 (21.3)
Total	61 (100.00)

### 3.3 Prevalence of Respiratory Symptoms

There were 6 respiratory symptoms that have been studied in order to find its association with occupational stress level. Examples of questions asked to the respondents were “Do you usually cough at all on getting up, or first thing in the morning?” and “Do you usually bring up phlegm at all on getting up or first thing in the morning?”. Table 4 shows the frequencies and percentages of respondents who have and did not have respiratory symptoms being studied. Symptoms that most experienced by the respondents were breathlessness with percentage of 47.5% followed by wheezing with percentage of 26.2%. 21.3% of the respondents had phlegm and followed by 14.8% of respondents experienced cough with phlegm and chest cold. Cough was experienced by 13.1% of the respondents.

**Table 4:** Respiratory symptoms among respondents (N=61)

Symptoms	N (%)	

	Yes	No
Cough	8 (13.1)	53 (86.9)
Phlegm	13 (21.3)	48 (78.7)
Cough with phlegm	9 (14.8)	52 (85.2)
Wheezing	16 (26.2)	45 (73.8)
Breathlessness	29 (47.5)	32 (52.5)
Chest cold & Chest illness	10 (16.4)	51 (83.6)

### 3.4 Association between Occupational Stress and Gender

Bivariate analysis has been conducted to find association between 2 categorical variables. Chi-square Test was used to determine association between occupational stress and gender. Table 5 shows the result of Chi-square Test. P-value obtained was 0.035 (χ^2^= 4.43) which shown significance association between occupational stress level and gender difference. Then, Odds Ratio (OR) obtained (OR= 4.14, 95% CI= 1.03 – 16.55) showed that female respondents was 4 times likely in getting occupational stress compared to male respondents.

**Table 5:** Association between occupational stress and gender (N= 61)

Occupational stress	Gender		χ^2^ value	p-value	CI (95%)	OR

	Female	Male				
High strain	13 (36.1)	3 (12.0)	4.433	0.035*	1.038 – 16.555	4.145
Low strain	23 (63.9)	22 (88.0)				

Chi-square Test

* Significant p < 0.05

### 3.5 Association between Occupational Stress and Gender

Chi-square Test and Fisher’s Exact Test were used to determine association between respiratory symptoms and gender. Table 6 shows the results of bivariate analysis conducted. The p-value obtained shows there were no significant association between cough, phlegm, cough with phlegm, wheezing also chest cold and chest illness with gender. Breathlessness was the only symptom that had a significant association with gender difference with p-value of 0.011 (χ^2^= 6.48). Based on Odds Ratio obtained (OR= 4.04, 95% CI= 1.34 – 12.14), female also was 4 times likely to get breathlessness compared to male respondents.

**Table 6:** Association between respiratory symptoms and gender (n=61)

Respiratory symptoms		Percentage of respondents (%)		χ^2^ value	p-value	CI (95%)	OR

		Female (N = 36)	Male (N = 25)				
Breathlessness	Yes	22 (61.1)	7 (28.0)	6.486	0.011*	1.344– 12.146	4.041
	No	14 (38.9)	18 (72.0)				
Phlegm	Yes	7 (19.4)	6 (24.0)	0.183	0.669	-	-
	No	29 (80.6)	19 (76.0)				
Cough with phlegm	Yes	5 (13.9)	4 (16.0)	-	1.000**#**	-	-
	No	31 (86.1)	21 (84.0)				
Wheezing	Yes	11 (30.6)	5 (20.0)	0.850	0.357	-	-
	No	25 (69.4)	20 (80.0)				
Cough	Yes	7 (19.4)	1 (4.0)	-	0.125**#**	-	-
	No	29 (80.6)	24 (96.0)				
Chest cold & chest illness	Yes	22 (88.0)	3 (12.0)	-	0.505**#**	-	-
	No	14 (12.0)	22 (88.0)				

Chi-square Test

# Fisher’s Exact Test

* Significant p < 0.05

### 3.6 Association between Socio-demography Factors and Occupational Stress

Association between socio-demography data and occupational stress level also been studied. Table 7 shows that there was no significant association between age (p= 0.819), marital status (p= 0.566), education level (p= 0.262), number of family members supported (p= 1.000) and working experience (p= 1.000) with occupational stress. Gender was the only factors that significantly associated with occupational stress with p= 0.035 (χ^2^= 4.43).

**Table 7:** Association between socio-demography factors and occupational stress (N=61)

Socio-demography factors	Occupational stress n (%)		χ^2^ value	p-value	OR	CI (95%)

	High strain	Low strain				
**Age**						
28 – 44	8 (50.0)	21 (46.7)	0.053	0.819	-	-
45 – 61	8 (50.0)	23 (53.3)				
**Gender**						
Female	13 (81.2)	23 (51.1)	4.433	*0.035	4.15	1.04– 16.56
Male	3 (18.8)	22 (48.9)				
**Marital status**						
Single	1 (6.2) 15	3 (6.7) 39		0.566		
Married	15 (93.7)	39 (86.7)	1.137		-	-
Divorced/widowed	0 (0.0)	3 (6.6)				
**Education level**						
Degree	1 (6.3)	0 (0.0)	-	0.262#	-	-
Master/PhD	15 (93.7)	45 (100.0)				
**Number of family members supported**						
0 - 4	13 (81.3)	36 (80.0)	-	1.000#	-	-
5 - 9	3 (18.7)	9 (20.0)				
**Working experience**						
4 - 25	13 (81.3)	37 (82.2)	-	1.000#	-	-
26 - 47	3 (18.8)	8 (17.8)				

Chi-square Test

# Fisher’s Exact Test

* Significant p < 0.05

### 3.7 Association between Occupational Stress and Respiratory Symptoms

Table 8 shows the association between high occupational stress level and respiratory symptoms being studied. From total number of 61 respondents, only 16 of them (26.2%) were classified under having high occupational stress level including male and female respondents. There were no significance association between occupational stress level with cough (p= 0.422), phlegm (p= 0.482), cough with phlegm (p= 1.000), wheezing (p= 0.322), breathlessness (0.163) and chest cold and chest illness (p= 0.686).

**Table 8:** Association between occupational stress and respiratory symptoms (N=61)

Symptoms		Occupational stress n (%)		χ^2^ value	p-value

		High strain	Low strain		
**Cough**	Yes	3 (18.8)	5 (11.1)	-	0.523#
	No	13 (81.2)	40 (88.9)		
**Phlegm**	Yes	3 (18.8)	13 (28.9)	-	0.482#
	No	13 (81.2)	32 (71.1))		
**Cough with phlegm**	Yes	2 (12.5)	7 (15.6)	-	1.000#
	No	14 (87.5)	38 (84.4)		
**Wheezing**	Yes	6 (37.5)	11 (24.4)	-	0.322#
	No	10 (62.5)	34 (75.6)		
**Breathlessness**	Yes	10 (62.5)	19 (42.2)	1.946	0.163
	No	6 (37.5)	26 (57.8)		
**Chest cold & chest illness**	Yes	5 (31.2)	7 (15.6)	-	0.270#
	No	11 (68.8)	38 (84.4)		

Chi-square Test

# Fisher Exact Test

## 4. Discussion

To determine occupational stress level among respondents, Job Content Questionnaires were used. Four factors have been considered in determining occupational stress level among respondents which were decision latitude, psychological job demand, job insecurity and social support. The same factors also have been studied by Azlihanis et al. (2006) in determining prevalence of occupational stress among secondary school teacher in Kelantan. Decision latitude was the ability to use his or her skills on the job and have authority to make decision regarding how the work was done and to set the schedule for completing work.

Psychological job demand was basically the psychological aspects of the job that require sustained physical and/or psychological effort. Although job demands were not necessarily negative, they may turn into job stressors when meeting those demands requires high effort (Wilmar et al., 2004). Following Karasek-Theorell Job Strain Model, decision latitude gave huge effect to the level of occupational among workers in the condition where lower score of decision latitude and high score of psychological job demand will result high occupational stress level and vice versa. Among 61 respondents, 16 of them (26.2%) experienced high occupational stress level which indicates that they were having low score of decision latitude and high score of psychological job demand which caused them unable to cope with stress from workplace. The prevalence was lower than study by Azlihanis et al. (2006) and Wei Sun et al. (2011).

Respiratory symptoms among the respondents were obtained by using American Thoracic Society Questionnaires. 6 respiratory symptoms being studied were cough, phlegm, cough with phlegm, wheezing, breathlessness, and chest cold & chest illness. Respiratory symptoms were studied in this study in order to find any association between occupational stress level and respiratory symptoms. It was postulated that stress can contribute to the development of diseases and infection in human body. This was due to the relation between stress and our immune system. The immune system was a collection of billions of cells that travel through the bloodstream. They move in and out of tissues and organs, defending the body against foreign bodies (antigens), such as bacteria, viruses and cancerous cells. When we were stressed, the immune system’s ability to fight off antigen was reduced. It would then make our body more susceptible toward infections (McLeod, 2010).

The stress hormone corticosteroid or also known as cortisol can suppress the effectiveness of the immune system (e.g. lowers the number of lymphocytes). In a classical study conducted by Kiecolt-Glaser et al. (1984), it was investigated that whether stress of important examinations has an effect on the functioning of the immune system by using numbers of T-cell lymphocytes as the indicator of immune system changes caused by stress. Researcher found that blood sample taken before the exam contained more T-cells compared with blood samples taken during the exam. It was also found that immune responses were especially weak in those respondents who reported feeling most lonely, as well as those who were experiencing other stressful life events and psychiatric symptoms such as depression and anxiety.

Symptoms that most experienced by the respondents were breathlessness with percentage of 47.5%. Most of the respondents who felt breathlessness agreed that they were troubled by shortness of breath when hurrying on the level or walking up a slight hill. Wheezing was the second symptoms most experienced by the respondents with the percentage of 26.2%. According to the questionnaires, respondents felt wheezing when they were having cold. Most of the respondents presented with the symptom for an average of 2.8 years. Phlegm was the third most experienced respiratory symptom with the percentage of 21.3 % from total respondents. Most of the respondents agreed that they usually brought up phlegm on getting up or first thing in the morning. Average of years for the respondents who troubled with phlegm was 2.7 years. 16.4% of respondents had chest cold and chest illness and also cough with phlegm gave percentage of 14.8%. All respondents had a period or episodes of cough with phlegm lasting for 3 weeks in each year with average of 3.8 years with the longest episodes of 10 years. For chest cold and chest illness, all 10 respondents agreed that when they got a cold, it would usually affect their chest and during past 3 months, some of the respondents need to off work and being indoor at home when having chest illness. Besides that, there were also few respondents did produce phlegm together while experiencing chest illness. The least symptom experienced by the respondents was cough where only 13.1% of the respondents had it. Most of the respondents usually cough on getting up or first thing in the morning. Also, some of them cough during the rest of the day or at night.

From the total of 25 male respondents, only 3 of them experienced high level of occupational stress and among 36 female respondents, 13 of them experienced high level of occupational stress. From Chi-square Test, p-value obtained was 0.035 which shown that there was a significant association between occupational stress level and gender difference. Odds Ratio obtained (OR= 4.14, 95% CI= 1.03 – 16.55) also indicate that female respondents had 4 times higher risk to experience high occupational stress level. Hypothesis for the fourth objective was not rejected. This finding was supported by a review by Steyn et al. (2006) that reported women has significantly higher level of stress compared to man. This was because women were often expected to meet other commitments, and conflicting work and family demands may add to their stressful response. However, this finding was contradicted with findings in study by Sun et al. (2011) among university teachers in China that the level of occupational stress was higher in male than in female even the finding was not significant with consideration of other factors.

As female respondents experienced more stress compared to male, hypothesis was made to evaluate that if the occupational stress level was high among female, then respiratory symptom should also high among female respondents. Comparison has been made using Chi-square test and Fisher’s Exact Test. All respiratory symptoms except breathlessness had no significance association with gender difference. For breathlessness symptom, from 25 male respondents, 7 respondents had the symptom and among 36 female respondents, 22 of them had the symptoms. From Chi-square Test, p-value obtained was 0.011 which shown that there was a significance difference between the symptoms and gender. Based on Odds Ratio obtained (OR= 4.04, 95% CI= 1.34 – 12.14), it indicates that females respondents were 4 times likely to experience breathlessness symptoms compared to male respondents. Hypothesis was not rejected. In a study on gender differences in respiratory symptoms by Dimich-Ward et al. (2006), women constantly had greater respiratory morbidity for symptoms associated with shortness of breath compared to male. According to the comprehensive US National Health Interview survey, (Lucas et al., 2004), women typically report more symptoms, more acute conditions, more days of restricted activity due to illness, and more doctor visits than men. For many diseases including respiratory conditions, population health surveys show that women tend to have greater physiological and psychological morbidity in comparison to men (Waldron, 1983).

Socio-demography factors that were taken into consideration in this study were age, gender, marital status, number of family members supported, education level and working experiences. All factors showed no significant association with high level of occupational stress except gender factor where there was a significant association between gender and occupational stress level with p-value of 0.035. This result was supported by study by Wei Sun et al. (2011), that sex or gender had been linked to occupational stress. Hypothesis for this objective was not rejected. In a study among educators of a secondary school, there was also no significant association between age, marital status, education level and working experience with occupational stress level (Azizi et al., 2004).

To determine the association between occupational stress level and respiratory symptoms, bivariate analysis has been performed. 16 respondents with high occupational stress level have been associated with 6 respiratory symptoms being studied. Table 4.8 shows the result of bivariate analysis. All p-value showed that there were no significant association between high level of occupational stress symptoms and all 6 respiratory symptoms being studied. DiCecco (2004) reported that in her study on daily hassles and respiratory symptoms intensity among college young adults, a weak correlation was found between stress and symptoms intensity, however the relationship was not significant. In contrast, several classical studies show that there was a relationship between psychological stress and respiratory symptoms such as cold (Cohen et al., 1991; Jacobs et al., 1970).

## 5. Conclusion

This study reported that among 61 lecturers being involved as the respondent, only 16 of them were having high level of occupational stress. While, the rest of the respondents fall under low strain group. Because of that, it can be concluded that, this group of lecturers of Universiti Putra Malaysia did not experienced high occupational stress level. Occupational stress level was not statistically significantly associated with all respiratory symptoms being studied.

## 6. Recommendation

As the results shows that more female lecturers experienced high occupational stress level compared to male, several ways were recommended in order to help reducing the stress. Firstly, lecturers need to have a good time management. They should be able to manage time wisely for example by having a planned schedule ahead for their daily routine and additional activities. By following the schedule, works can be done on time and there would be no work abundance that can contribute to occupational stress. Along with responsibilities at workplace, female lecturers would also need to handle household chores and spending some times for their husband and children. Thus, dividing their time appropriately can give a balance space for each responsibility.

Besides that, female lecturers with high occupational stress level were suggested to attend stress coping program in order to learn how to cope with stress in their daily life. Consultation with counselor was also a good choice as they can teach them directly how to deal with stress. Stress can be also higher among female lecturers who had children at home. So, it was recommended that an institution should have a nursery at or nearby the office to reduce worriedness of their workers regarding their children welfare.

Response rate of the study shows that less than 50% of the lecturers willing to be involved in the study as some of them rejected the invitation of the study due to several factors. Firstly, lecturers refused to be involved in this study due to time constraint. As study was conducted two weeks before the new semester started, they might need to fully use their working time to finish their work and therefore did not enough time to answer the questionnaires. Besides that, they might have abundant of works to be finished and settled before student admission. Therefore, representativeness of the sample toward study population cannot be ensured. Also, this study was conducted only among Universiti Putra Malaysia and did not involve lecturers from other academic institution. Author suggest that in the future study, it should be made compulsory to all lecturers of respected universities or institution to be involved in study conducted as it will be beneficial to both respondents and researchers.

Since there was only limited study on association between occupational stress and respiratory symptoms among university’s lecturers locally, it is suggested that more future study to be carried out with larger sample of respondents to obtain more precise, comprehensive and representative results. Researchers may expand the scope of this research and studies more details on the mechanism and effects of stress towards human body.
